# NF-κB Links TLR2 and PAR1 to Soluble Immunomodulator Factor Secretion in Human Platelets

**DOI:** 10.3389/fimmu.2017.00085

**Published:** 2017-02-06

**Authors:** Pauline Damien, Fabrice Cognasse, Bernard Payrastre, Sherry L. Spinelli, Neil Blumberg, Charles-Antoine Arthaud, Marie-Ange Eyraud, Richard P. Phipps, Archibald McNicol, Bruno Pozzetto, Olivier Garraud, Hind Hamzeh-Cognasse

**Affiliations:** ^1^GIMAP-EA3064, Université de Lyon, Saint-Étienne, France; ^2^Etablissement Français du Sang Rhône-Alpes-Auvergne, Saint-Etienne, France; ^3^Inserm, U1048 and Université Toulouse 3, I2MC, CHU de Toulouse, Laboratoire d’Hématologie, Toulouse, France; ^4^Department of Pathology and Laboratory Medicine, University of Rochester School of Medicine and Dentistry, Rochester, NY, USA; ^5^Faculty of Pharmacy, University of Manitoba, Winnipeg, MB, Canada; ^6^Institut National de Transfusion Sanguine (INTS), Paris, France

**Keywords:** platelets, NF-κB, phosphorylation, TLR2, cytokine/chemokine, inflammation

## Abstract

The primary toll-like receptor (TLR)-mediated immune cell response pathway common for all TLRs is MyD88-dependent activation of NF-κB, a seminal transcription factor for many chemokines and cytokines. Remarkably, anucleate platelets express the NF-κB machinery, whose role in platelets remains poorly understood. Here, we investigated the contribution of NF-κB in the release of cytokines and serotonin by human platelets, following selective stimulation of TLR2 and protease activated receptor 1 (PAR1), a classical and non-classical pattern-recognition receptor, respectively, able to participate to the innate immune system. We discovered that platelet PAR1 activation drives the process of NF-κB phosphorylation, in contrast to TLR2 activation, which induces a slower phosphorylation process. Conversely, platelet PAR1 and TLR2 activation induces similar ERK1/2, p38, and AKT phosphorylation. Moreover, we found that engagement of platelet TLR2 with its ligand, Pam3CSK4, significantly increases the release of sCD62P, RANTES, and sCD40L; this effect was attenuated by incubating platelets with a blocking anti-TLR2 antibody. This effect appeared selective since no modulation of serotonin secretion was observed following platelet TLR2 activation. Platelet release of sCD62P, RANTES, and sCD40L following TLR2 or PAR1 triggering was abolished in the presence of the NF-κB inhibitor Bay11-7082, while serotonin release following PAR1 activation was significantly decreased. These new findings support the concept that NF-κB is an important player in platelet immunoregulations and functions.

## Introduction

Besides their central role in hemostasis, platelets have now entered the field of immunity as inflammatory cells (1–5). Initial studies reported the first evidence of the presence of toll-like receptors (TLRs) on human platelets and showed that their level significantly increases following activation (6, 7). We also found that stored platelets contain molecules with known immunomodulatory roles and differentially secrete them upon storage in blood banking conditions (8–11). One of the best-studied platelet TLRs is TLR4. Recent studies by our group (12) and others (13, 14) support the idea that platelets are able to distinguish between membrane signaling molecules and can for instance, adapt their secretion to specific signals sensed *via* TLR4. Recently, we have shown that soluble (s)CD14 from plasma contributes to LPS/TLR4 signaling in platelets to allow the potent release of sCD40L, thereby elucidating the mechanism of LPS-induced platelet responses and providing new insights for reducing LPS toxicity in the circulation (15).

Toll-like receptor 2 is also expressed in platelets and recognizes components of Gram-positive bacteria. In cooperation with TLR1 and TLR6, TLR2 recognizes bacterial lipoproteins and contributes to the formation of thrombi (16). Several recent studies have shown that TLR2 is involved in platelet signal transduction, platelet aggregation, adherence to collagen, and formation of platelet–neutrophil heterotypic aggregates (16–20). Moreover, a procoagulant phenotype of extracellular histone-activated platelets driving plasma thrombin generation has been reported by Semeraro et al., who suggested potential roles for TLR2 and TLR4 in this process (21).

As platelets are anucleate, the role of transcription factors downstream of their surface receptors remains unclear, but of considerable fascination. The NF-κB signaling machinery is present in platelets (22–24), but its role downstream of TLR2 and TLR4 remains poorly characterized.

Recent data suggest that NF-κB has unexpected roles in regulating non-genomic processes involved in platelet functions (13, 14, 22, 23, 25) and show that NF-κB inhibitors affect platelet functions suggesting different effects of the NF-κB machinery in platelets (23, 25).

The innate immune system senses proteolytic enzymes generated during infection through a family of classical PRRs, such as TLRs, and “non-classical” PRRs, for example, PARs (26, 27). TLR4 engagement has been investigated in several ways [including by ourselves (6, 12, 15, 28–30)]. The aim of our study was to compare the effects of TLR2 stimulation on several protein phosphorylation processes and to link these effects to platelet alpha granule protein and serotonin release. To achieve this goal, we compared engagement of TLR2 and the classical thrombin receptor, protease activated receptor 1 (PAR1), on platelet function.

## Materials and Methods

### Platelet Preparation and Stimulation

Peripheral blood was collected from healthy donors in endotoxin-free 3.2% sodium citrate tubes (Vacutainer^®^, Becton Dickinson, San Jose, CA, USA) within the French National Blood Establishment fulfilling the national principles of ethics and the regulatory requirements. Platelet-rich plasma (PRP) was prepared as described previously (15).

Platelet-rich plasma (3 × 10^8^ platelets/ml) were incubated for 30 min at room temperature (RT) with the anti-human FcγRII monoclonal antibody (MoAb) IV.3 (100 µg/ml; StemCell Technologies, Grenoble, France) in order to saturate free FcγRII sites and block any FcγRII engagement (30, 31). Next, platelets were stimulated with the TLR2 ligand Pam3CSK4 (Cayla-Invivogen, Toulouse, France; 1–100 µg/ml, 120 min, RT) in the absence or presence of either one or two anti-human TLR2 blocking MoAbs, clone TL2.1 (8 µg/ml, 30 min, RT; Imgenex, San Diego, CA, USA), and clone B4H2 (8 µg/ml, 30 min, RT; Cayla-Invivogen) or with an irrelevant specificity MoAbs IgG2a (BD Biosciences, Le Pont de Claix, France) control (16). Thrombin receptor activator peptide (TRAP; 50 µg/ml; Sigma-Aldrich, Saint Quentin Fallavier, France), an agonist of PAR1, was used as a positive control.

### Platelet Phosphoprotein Profile

pNF-κB (phosphorylated NF-κB at S536 and S468 on p65 subunit) and total p65 subunit levels in extracts from stimulated versus non-stimulated platelets were determined by enzyme-linked immunosorbent assays (ELISA). pNF-κB (assay sensitivity = 0.5 µg nuclear extract/well) was detected using a transcription factor ELISA kit (Active Motif), as previously described (32). Protein phosphorylation of ERK (pT185/pY187), p38 (pT180/pY182), and Akt (pS473) was determined using the 9-plex Multi-Pathway Magnetic Bead Panel (Millipore #46-680MAG, Amsterdam, Netherlands) following the manufacturer’s instructions with the Median Fluorescence Intensity measured by the Luminex System.

### Flow Cytometry and Western Blot

Expression of TLR2 on the platelet surface was determined by flow cytometry analysis of all events positive for CD41, a characteristic platelet surface marker. Platelets were labeled with a PE-conjugated anti-human TLR2 MoAb (Imgenex) or CD62P MoAb (BD Biosciences). Positive events were recorded with a FACSCalibur (BD Biosciences) flow cytometer and analyzed with CELL QUEST PRO software from BD Biosciences.

Western blot analysis of the expression of TLR2 (3 µg/ml; Imgenex), MyD88 (3 µg/ml, Santa Cruz Biotechnology), NFκB p65 (1 µg/ml; Santa Cruz Biotechnology), histone H3 (diluted 1/1,000; Abcam), or α-tubulin (200 ng/ml; Santa Cruz Biotechnology) was prepared as described previously (28).

### Enzyme-Linked Immunosorbent Assay for Soluble Factors in Platelet Supernatants

The levels of soluble cytokines sCD62P, RANTES, PF4, and sCD40L were measured in triplicate from aliquots of unstimulated (control) and TLR2 ligand-stimulated platelets using specific ELISAs (R&D Systems Europe Ltd., Lille, France or Abcyss, Paris, France). The serotonin levels were measured by competitive ELISA according to the manufacturer’s instructions (IBL Immuno-Biological Laboratories, Hamburg, Germany) as previously described (33).

### Analysis of Signaling Pathway Activation

Platelets were pre-incubated with various concentrations of the non-reversible inhibitor of NF-κB, Bay 11-7082 (2 μM) (Sigma-Aldrich) or Ro 106-9920 (4 μM) (Tocris Bioscience), for 60 min at 37°C (23). Platelets were then stimulated with Pam3CSK4 or TRAP in the presence or absence of an anti-human TLR2 blocking MoAb to detect NF-κB signal modulation. Platelet supernatants were collected to determine soluble factors levels as outlined above.

### Statistical Analysis

All values are reported as mean ± SD. Almost all measurements were non-normally distributed. Non-normally distributed data were analyzed using non-parametric tests: Mann–Whitney *U* test. Results are given as means with medians with ranges (non-parametric tests). Statistical test differences were considered significant if *P* values were less than 0.05.

## Results

### Platelets Express Components of the TLR2 Signaling Complex

We first tested for the expression of TLR2 on the surface of human blood platelets and the presence of its potential downstream signaling machinery. TLR2 is expressed on the surface of resting platelets, as shown by flow cytometry (Figure S1 in Supplementary Material), at a mean percentage of 30.1 ± 3.76%. Supporting this finding from others (20), we observed that activation of human platelets with TRAP did not significantly affect the baseline expression of TLR2; TRAP cannot thus be ascribed as over regulating TLR2 expression.

Western-blotting analysis (Figure S1 in Supplementary Material) confirmed not only the expression of TLR2 but also, as expected, MyD88 and NF-κB in human platelets (16, 19, 22, 23, 25, 34). The mononuclear cell level in platelet preparations was undetectable, as determined by flow cytometry (15). The nuclear marker histone H3 was also undetectable in our platelet preparation (28), consistent with the absence of nuclear factors originating from potential contaminating nucleated cells. Taken together, these data support that a significant population of resting platelets express TLR2 on their surface and have the potential downstream effectors to mediate TLR2 signaling.

### Effect of TLR2 and PAR1 Stimulation on Platelet Phosphoprotein Profiles

**Figure 1:**
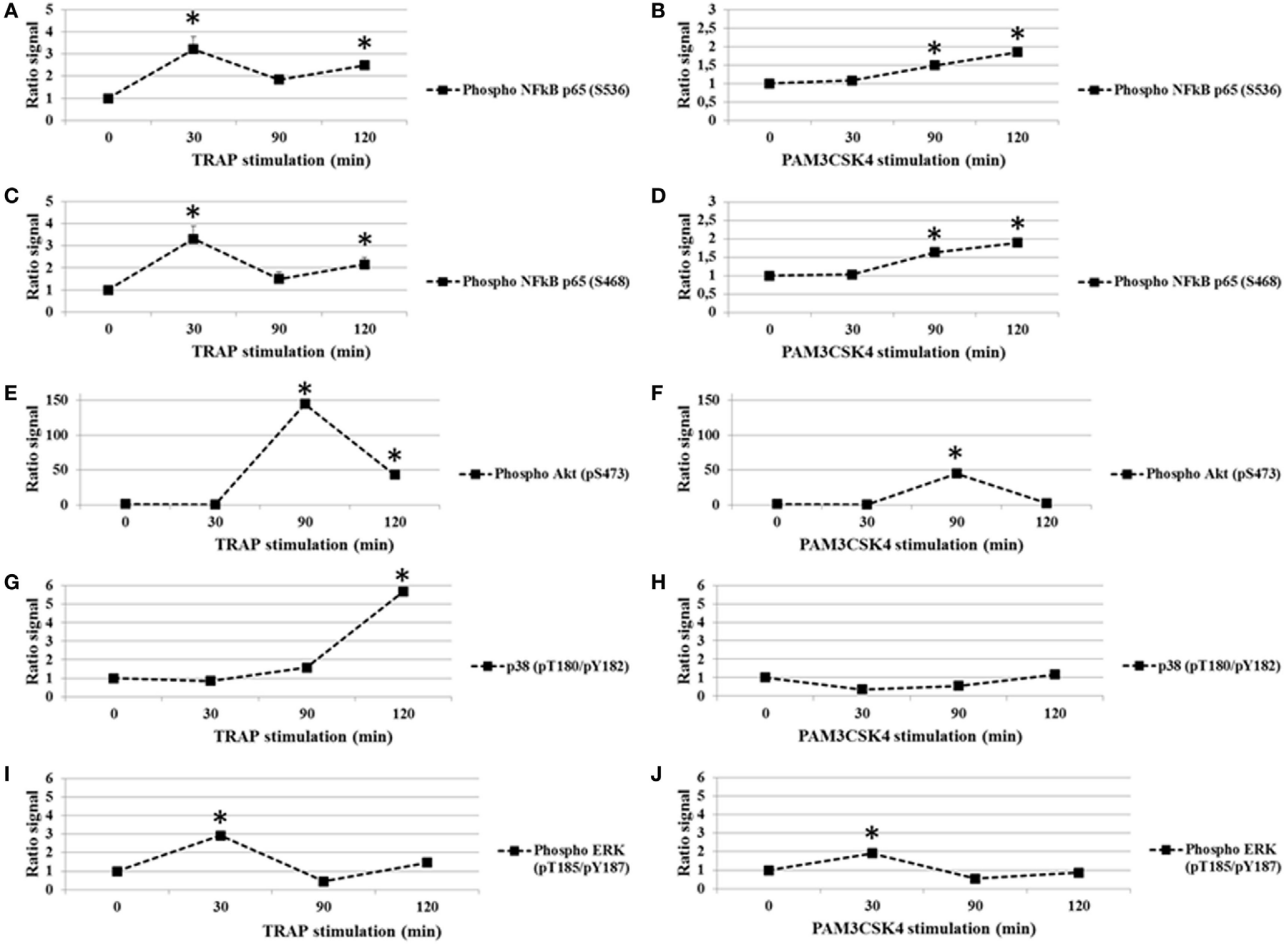
**TRAP and toll-like receptor 2 stimulation in platelets induces changes in phosphorylation (ratio stimulated/unstimulated signal)**. Analysis of p65 subunit of NF-κB phosphorylated S536 **(A,B)** and S468 **(C,D)**, Akt (pS473) **(E,F)**, P38 (pT180/pY182) **(G,H)**, and ERK1/2 (pT185/pY187) **(I,J)** in platelet extracts from TRAP (50 µg/ml) or Pam3CSK4 (100 µg/ml) stimulated versus non-stimulated platelets. The analysis was performed at different times from 0 to 120 min of stimulation by TRAP (50 µg/ml) **(A,C,E,G,I)** or Pam3CSK4 (100 µg/ml) **(B,D,F,H,J)**. Data are mean (± SD) of five independent experiments (measured in triplicate). *P < 0.05 (Mann–Whitney *U* test; stimuli versus unstimulated).

In addition to nuclear translocation, the phosphorylation of the p65 subunit of NF-κB on specific serine residues impacts its activity. In particular, the phosphorylation of serine 536 (S536) and serine 468 (S468) are important for p65-induced cellular responses (35). We first tested whether platelet activation by TRAP (0–120 min) induced the phosphorylation of the p65 at residues S536 and S468. As shown in Figures 1A,C, while total p65 protein levels remained stable (data not shown), there was a rapid and significant increase in S536 and S468 phosphorylation following the addition of TRAP. The phosphorylation was dose-dependent, becoming detectable between 50 and 250 ng/ml of TRAP (data not shown). The maximal phosphorylation of both S536 (Figure 1A) and S468 (Figure 1C) was observed at 30 min of stimulation. Thereafter, phosphorylation levels decreased to near basal levels by 90 min, but had increased again at 120 min, suggesting the existence of a second wave of phosphorylation (Figures 1A,C). Oscillatory dynamics of NF-κB nuclear translocation and phosphorylation has been described in nucleated eukaryotic cells (36). To our knowledge, this has not been reported previously for anucleate cells, such as platelets. Interestingly, when human platelets were stimulated with the TLR2 agonist Pam3CSK4 (100 µg/ml), the level of S536 phosphorylation (Figure 1B) and S468 (Figure 1D) remained low until 30 min and then significantly increased until the end of the time course (120 min).

Because Akt, P38, and ERK1/2 are known to affect the modulation of platelet function and several data support a strong mechanistic-link between the NF-κB, Akt, P38, and ERK1/2 pathways (37–41), we analyzed the effect of PAR1 or TLR2 stimulation on these platelet phosphoprotein profiles (Figures 1E–J). Platelets were exposed to TRAP (50 µg/ml) or PAM3CSK4 (100 µg/ml) for increased times (0–120 min), and these intracellular signaling factors were evaluated. First, we demonstrate that phosphorylation levels of Akt (pS473), p38 (pT180/Y182) and ERK1/2 (pT185/Y187) change over time in response to stimulation by either TRAP or Pam3CSK4 (Figures 1E–J). Second and in contrast to our findings for p65 phosphorylation, TRAP and Pam3CSK4 elicit similar patterns of phosphorylation for Akt (Figures 1E,F), p38 (Figures 1G,H), and ERK1/2 (Figures 1I,J), although the kinetics of phosphorylation are distinct for each phosphoprotein. Maximal phosphorylation levels are observed at 90, 120 and 30 min, respectively. Overall, these results demonstrate that a highly regulated protein phosphorylation network exists in human platelets.

### Platelet Soluble Immunomodulatory Factors Are Released after TLR2 or PAR1 Engagement

**Figure 2:**
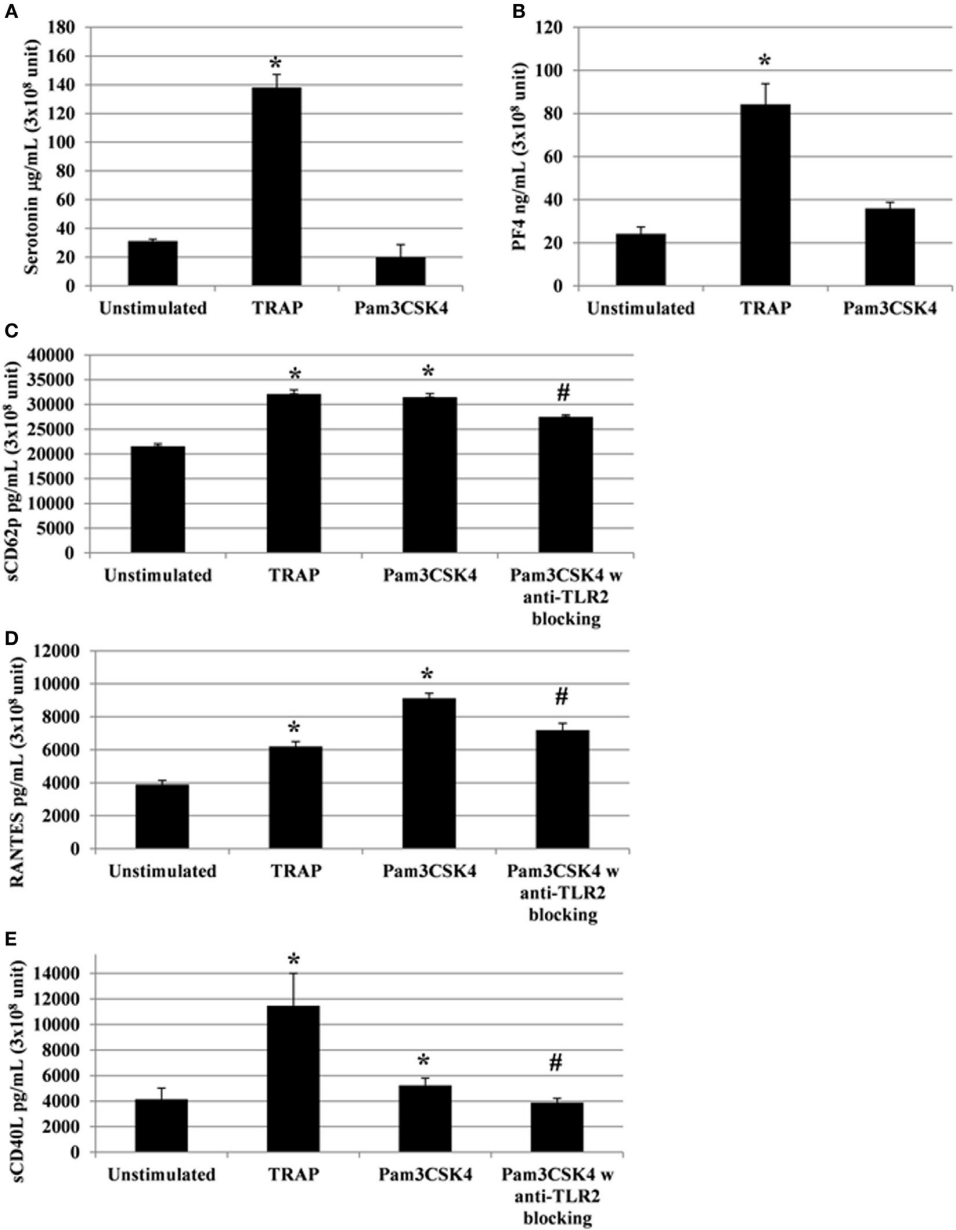
**Platelets release soluble immunomodulatory factors following toll-like receptor (TLR) 2 or protease activated receptor 1 engagement**. Platelet serotonin, sCD62P, RANTES, and sCD40L are released by platelets stimulated by TRAP (50 µg/ml) or Pam3CSK4 (100 µg/ml) with (w) or without (w/o) anti-human TLR2 blocking monoclonal antibody (MoAb). Platelets were pre-treated (20 min, room temperature) with anti-human FcγRII MoAb IV.3 and incubated with TRAP or Pam3CSK4 for 120 min. The levels of serotonin **(A)**, PF4 **(B)**, sCD62P **(C)**, RANTES **(D)**, and sCD40L **(E)** were quantified by enzyme-linked immunosorbent assays (measured in triplicate). Background levels were subtracted from the values shown. Data are mean ± SD (*n* = 5 experiments, measured in triplicate) and are expressed as picograms per milliliter (3 × 10^8^ unit—sCD62P, RANTES, and sCD40L) or as micrograms per milliliter (3 × 10^8^ unit—serotonin). The soluble factors values shown are deduced from background levels. *P < 0.05 (Mann–Whitney *U* test; stimuli versus unstimulated). Anti-human TLR2 blocking MoAb (α-TLR2) was used at a concentration of 8 µg/ml. ^#^*P* < 0.05 (Mann–Whitney *U* test; stimuli w anti-TLR2 block versus stimuli w/o anti-TLR2 block).

Following the addition of conventional platelet agonists, such as thrombin or collagen, release of cytokines/chemokines occurs between 5 and 30 min (42). A titration and time course were performed using human platelets collected from healthy donors to determine the optimal concentration of Pam3CSK4 platelet activation, using sCD62P and RANTES release as end points (Figure S2 in Supplementary Material). We, then, monitored platelet cytokine/chemokine secretion for 2 h following TLR2 stimulation by Pam3CSK4 (100 µg/ml). TRAP (50 µg/ml) was also used for comparison, as it potently induces platelet release of cytokines/chemokines. In contrast to stimulation by TRAP, Pam3CSK4 had no significant effect on serotonin release (Figure 2A), nor PF4 release (Figure 2B; Figure S6 in Supplementary Material). In contrast, Pam3CSK4 stimulation significantly increased the production of sCD62P, RANTES, and sCD40L (Figures 2C–E, respectively). In contrast, the production of each of these mediators was significantly attenuated when platelets were pre-incubated for 30 min at RT with an anti-TLR2 MoAb (Figures 2C–E).

Importantly, to avoid any potential effect of the Fc portion of the anti-TLR2 antibody on FcγRIIA, the IV.3 blocking antibody was used in our experiments. As shown in Figure S3 in Supplementary Material, the addition of IV.3 antibody had no effect on platelet secretion. Moreover, there was no significant modification of sCD62P, sCD40L, RANTES, and serotonin release after stimulation in the presence of isotype-matched, irrelevant specificity MoAbs IgG2a (Figure S4 in Supplementary Material). Overall, these results demonstrate that TLR2 triggering results in the secretion of a set of platelet cytokine/chemokine independently of FcγRIIA.

### Relationship between Specific TLR2 Stimulation and NF-κb Transducer Molecules

**Figure 3:**
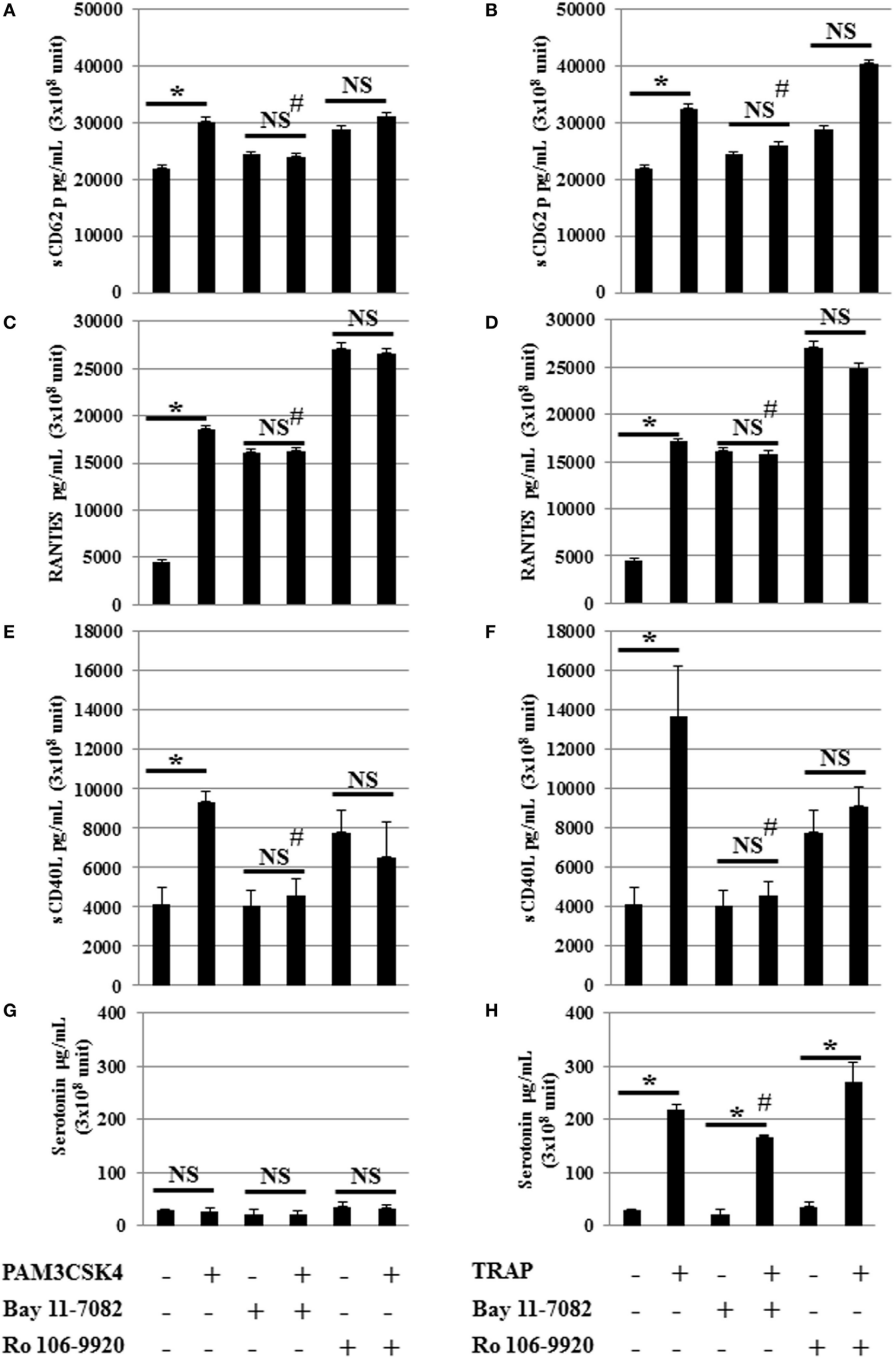
**NF-κB inhibition attenuates toll-like receptor 2 and protease activated receptor 1 stimulated mediator release**. Serotonin, sCD62P, RANTES, and sCD40L secretion levels from platelets stimulated by Pam3CSK4 (100 µg/ml) **(A,C,E,G)** or TRAP (50 µg/ml) **(B,D,F,H)** with (w) or without (w/o) the inhibitor of NF-κB (BAY 11-7082). Platelets were pre-treated (20 min, room temperature) with anti-human FcγRII monoclonal antibody IV.3 and incubated with inhibitor and stimulators as described in Section “Materials and Methods.” Levels of sCD62P **(A,B)**, RANTES **(C,D)**, sCD40L **(E,F)**, and serotonin **(G,H)** were quantified by enzyme-linked immunosorbent assays (measured in triplicate). Background levels have been subtracted and the data (mean ± SD; *n* = 10 experiments, measured in triplicate) are expressed in picograms per milliliter. *P < 0.05; Mann–Whitney *U* test, unstimulated without inhibitor (BAY 11-7082) versus stimulated without inhibitor (BAY 11-7082) or unstimulated in the presence of inhibitor (BAY 11-7082, 2 μM) versus stimulated in the presence of inhibitor (BAY 11-7082, 2 μM). ^#^*P* < 0.05 (Mann–Whitney *U* test); stimulated without inhibitor (BAY 11-7082) versus stimulated in the presence of inhibitor (BAY 11-7082, 2 μM).

In nucleated cells, NF-κB p65 is an important downstream effector of TLR2 through the adapter protein MyD88 followed by NF-κB triggering of inflammatory cytokine production. To determine whether NF-κB is involved in the effects of TLR2 in platelets, we used an inhibitor of NF-κB suitable for *in vitro* studies (Figure 3). This compound is a highly selective and widely used irreversible inhibitor of NF-κB that impairs IκBα phosphorylation (23, 43). Interestingly, the release of sCD62p, RANTES, and sCD40L following TLR2 engagement by Pam3CSK4 was decreased to almost background levels in the presence of this NF-κB inhibitor (Figures 3A,C,E,G). Similar results were obtained following stimulation with TRAP (Figures 3B,D,F,H), suggesting that NF-κB is not exclusively required downstream of TLR2 to mediate cytokine secretion, but is also important downstream of the stimulation of PAR1. We observed a significant (# *P* < 0.05) decrease of the release of sCD62p, RANTES, and sCD40L following TRAP and Pam3CSK4 stimulation when comparing stimulated platelets without inhibitor (BAY 11-7082 or Ro 106-9920) versus stimulated in the presence of inhibitor (BAY 11-7082 or Ro 106-9920). Using an inhibitor of NF-κB suitable for *in vitro* studies BAY 11-7082 or Ro 106-9920 showed similar results. In contrast to TRAP, which induced platelet serotonin release, as expected, Pam3CSK4 stimulation did not (Figure 3G). Although NF-κB inhibition by BAY-11-7082 or Ro 106-9920 decreased both the basal level of serotonin and the amount of serotonin secreted upon TRAP activation, it did not significantly affect the ability of TRAP to stimulate serotonin secretion (4.45-fold increase in the absence of BAY-11-7082 versus 5.35-fold increase in the presence of BAY-11-7082) (Figure 3H).

## Discussion

Toll-like receptors expressed by various cell types are now well recognized as sentinels of the innate immune system, keeping vigilance against invading pathogens (44).

Platelets express several functional TLRs, such as TLR1, TLR2, TLR4, TLR7, and TLR9 (6, 20, 45–47). Recently, Tunjungputri et al. (48) demonstrated an association with an increase in pro-inflammatory cytokines in blood exposed to Pam3CSK4, but a decrease in these cytokines in blood exposed to LPS. These findings indicate that platelets differentially modulate TLR2- and TLR4-mediated cytokine responses of PBMC. We tested for platelet activation markers and sCD62P release following TLR2-, 4-, 7-, and 9-stimulation, and we evidenced different behaviors of platelets depending on the type of TLR that was engaged (Figure S5 in Supplementary Material). Our experimental unpublished data (Figure S5 in Supplementary Material) suggest that platelet TLR2, 3, 4, 7, and 9 are functionally active in inducing sCD62P in response to their specific ligands, although the effect on the soluble CD62P is differential. These data suggest that platelets have the capacity to sense external signals differentially through pathogen recognition receptors and adjust the innate immune response appropriately for pathogens exhibiting different types of “danger” signals.

Human platelets express TLR1 (7, 16) and Pam3CSK is a synthetic TLR2/TLR1 agonist, and therefore, we cannot exclude that TLR1 play a role in our model. Nevertheless, Blair et al. (16) demonstrate that neutralization of TLR2 significantly reduced Pam3CSK4-induced platelet aggregation and TLR1 inhibition also reduced aggregation, although not as effectively as TLR2 blockage.

Recent reports demonstrate that, despite being anucleate, platelets express a number of transcription factors, including the NF-κB transcription factor family (1, 23, 25, 49), retinoic X receptor and peroxisome proliferator-activated receptors (PPARγ and β/δ) (50, 51). In the present study, we investigated pathways considered essential for inflammatory cytokine production downstream of TLR2 stimulation (52) and found an important and new contribution by NF-κB in human platelets. In particular, we have demonstrated the differential mobilization of the NF-κB pathway following exposure of platelets to ligands of innate immunity receptors (Pam3CSK4 for TLR2) compared to ligands of the thrombotic stimulation (TRAP for PAR1). In eukaryotic cells, the transcription of target genes depends on oscillation persistence, involving cycles of RelA (p65) phosphorylation and dephosphorylation (36). Interestingly, changes in platelet p65 phosphorylation following TRAP stimulation were observed. The presence and non-genomic functions of NF-κB family members have been demonstrated by several groups (1, 23, 25, 38, 43, 53). Importantly, several reports suggest that platelets contain an intact, functional, and complete NF-κB pathway. Further, PAR1 has a lower threshold for activation by thrombin than PAR4. PAR1 activation typically induces a rapid, but transient spike in calcium, while PAR4 activation involves a more sustained response, suggesting overlapping, but distinct roles for these receptors. These data reveal a novel and distinct signaling pathway for PAR1 and PAR4 receptors, although both converge on NF-κB signaling. Taken together, these data present compelling evidence that NF-κB plays an important, albeit complex, role in platelet activation. Nevertheless, Lannan et al. (1) described that thrombin signaling through PAR1 in human platelets is mostly independent of NF-κB. These findings highlight the complexity of biological responses in platelet signaling. As with eukaryotic cells, computational models will prove to be useful in deciphering the complex signaling networks found in platelets. This study focused on NF-κB modulation concerning platelet activation in response to PAM3CSK4 and/or TRAP. However, the inhibition of NF-κB also affects platelet activation in response to other agonists as can be found in other reports (43, 53). Two specific unrelated inhibitors of NF-κB activation, BAY 11-7082 and Ro 106-9920, reduced PAC-1 and fibrinogen binding to integrin αIIbβ3 and restricted platelet spreading on immobilized fibrinogen. Both inhibitors impaired aggregation mediated by ADP, epinephrine, collagen, or thrombin, but not arachidonic acid. ATP release, TXB2 formation, CD62P expression, ERK phosphorylation, and cPLA2 activity stimulated by thrombin were reduced in BAY 11-7082- or Ro 106-9920-treated platelets (43). Moreover, platelet stimulation with Pam3CSK4 or LPS resulted in IκBα degradation and p65 phosphorylation. These responses were suppressed by TLR2 and 4 blocking. Aggregation, fibrinogen binding, and ATP and vWF release were triggered by Pam3CSK4. LPS did not induce platelet responses *per se*, except for vWF release, but it did potentiate thrombin-induced aggregation, fibrinogen binding and ATP secretion. Pam3CSK4, but not LPS, induced CD62P and CD40L expression and mixed aggregate formation. All of these responses, except for CD40L expression, were inhibited in platelets treated with the NF-κB inhibitors BAY 11-7082 or Ro 106-9920 (53).

There is indication that α-granule subpopulations can be identified on the basis of morphology, cargo type, and response to agonists (54). Contrary to Berg et al. (55) who described that Pam3CysSer(Lys)4, another lipopeptide analog of the NH2-terminus of bacterial lipoprotein used in our model, led to the aggregation of platelets and induced the secretion of serotonin with an effectiveness similar to thrombin. Here, we show that contrary to stimulation by TRAP, Pam3CSK4 had no significant effect on serotonin and PF4 release. In contrast, Pam3CSK4 stimulation highly increased the production of RANTES in the supernatant of PRP. Koenen et al. have shown that disruption of PF4-RANTES heterodimers by high affinity peptide ligands can slow the development of atherosclerotic plaque development in murine models of vascular disease (56). In addition, our results confirm that platelet secretion of selected immunomodulatory factors may be an important inflammatory-regulating mechanism.

In platelets, NF-κB/I-κB proteins have been described to function independently of gene regulation; after activation, IκB is phosphorylated and degraded (22). Recent new evidence suggests a potential novel pathway of platelet regulation by NF-κB, linked to key platelet responses (e.g., platelet spreading, aggregation, clot retraction) (23, 25).

Recently, Rivadeneyra et al. (53) described TLR2 and 4 agonists trigger platelet activation responses through NF-κB. Our data, thus, support the Rivadeneyra data and particularly the modulation of expression of platelet TLR2, as shown concomitantly in 2005 by our group (6) and the Semple group (20). We too observed that platelet TLR2 activation results in the activation of the NF-κB pathway. The pNF-κB (phosphorylated NF-κB at S536 and S468 on p65 subunit) and total p65 subunit levels in extracts from stimulated versus non-stimulated platelets were determined by ELISA and not by Western Blot, which allowed us to precisely demonstrate that p65 S536 and S468 subunits were mobilized. Moreover, our data show a process concerning phosphorylation of NF-κB in human platelets after TLR2 activation (with Pam3CSK4) and PAR-1 (TRAP), which has not previously been demonstrated in platelet signaling. Importantly, our findings reported here extend the Rivadeneyra data. We demonstrate that Pam3CSK4 or TRAP induction of NF-κB-mediated pro-inflammatory responses have differential effects with regard to the release of soluble factors in platelet supernatants, such as sCD62P, RANTES, PF4, and sCD40L, markers of α granule release, and serotonin, a marker predominantly present in dense granules. In contrast to stimulation by TRAP, Pam3CSK4 had no significant effect on serotonin nor PF4 release. Conversely, Pam3CSK4 stimulation significantly increased the production of sCD62P, RANTES, and sCD40L. Our findings support the hypothesis that TLR-2 and PAR-1 platelet stimulation triggers differential protein mobilization; however, further studies exploring physiological conditions are needed to draw firmer conclusions regarding this hypothesis.

It has been suggested that platelets selectively release their alpha and dense granule content dependent on the environmental stimulus. Several *in vitro* data revealed that different granule subsets are selectively released by specific agonists, as suggested by agonist-specific a-granule secretion kinetics (57–60). Similarly, Chatterjee et al. demonstrated that PAR1 stimulation or ADP exposure elicited robust release of stromal cell-derived factor1 and VEGF, but modest release of PF4 or endostatin (61). An explanation for these observations is that different cargos are segregated into different granule subpopulations. This observation was correlated with two other publications. Peters et al. (54) demonstrated that alpha granule can be distinguished based on the VAMP isoforms that they express and that a population of granules expressing VAMP-7 physically separated from other granule populations during spreading. Van Nispen tot Pannerden et al. used cryoelectron tomography to provide a 3-dimensional (3D) map of the intracellular membrane organization of human platelets and revealed a large heterogeneity in the platelet membrane organization. They concluded that aside from the existence of morphologically different alpha-granule subtypes, the 3D study demonstrated that high spatial segregation of cargo exists within individual alpha-granules (62).

Moreover, recent studies by Starlinger et al. present clinical evidence that the profile of circulating platelet a-granule proteins seems to affect postoperative liver regeneration in patients after liver resection. They conclude that selective a-granule release might modify pathophysiological processes involved in liver regeneration (63). Our observations support the concept that NF-κB proteins are important in both the pro-thrombotic and immunomodulatory functions of platelets. Indeed, NF-κB inhibition significantly decreased the secretion of serotonin, cytokines, and chemokines from platelets stimulated either by Pam3CSK4 or by TRAP, a PAR1 agonist. This is consistent with studies by Malaver et al. who showed that BAY-11-7082 inhibited platelet aggregation (43) and by Spinelli et al. who demonstrated that inhibition of I-κB-α phosphorylation by BAY-11-7082 caused reversal of fully spread platelets to their original spheroid morphology (23).

Our data also highlight the functional role of the TLR signaling pathway. This is important as platelets interact primarily with the vessel endothelium, a tissue that can be inflamed and is reactive to pro-inflammatory products originating from platelets (64). We recently proposed (30, 65) that platelets employ adaptive responses to danger signals, which involve a polarized secretion for soluble immunomodulatory factors; NF-κB could orientate this. The discovery of non-hemostatic functions for platelets continue to increase, and include the role of platelets in infectious and/or inflammatory diseases. Future work is necessary to elucidate whether TLR2 (and/or TLR1) activation has a direct effect on platelet activation, and to further define the role of platelets in innate immune signaling. Importantly, TLR2 (and/or TLR1) could represent a potential new target for drug discovery. However, in order to develop new pharmaceutical drugs that target platelet participation in infectious and/or inflammatory disease, a novel role for platelet TLR signaling must be demonstrated that is independent of prothrombotic pathways.

## Ethics Statement

Peripheral blood was collected from healthy donors in endotoxin-free 3.2% sodium citrate tubes (Vacutainer^®^; Becton Dickinson, San Jose, CA, USA) within the French National Blood Establishment fulfilling the national principles of ethics and the regulatory requirements. Briefly, peripheral blood were collected from regular anonymous blood donors (Regional Blood Bank, EFS Auvergne-Loire—http://www.dondusang.net) who volunteered to provide blood for research purposes and signed a consent form, approved by the ethical committees of Etablissement Français du Sang. Peripheral blood collection are identified with bar-codes and none of the investigators can reconcile any single donor and his/her given peripheral blood collection (only the blood service physician can in case of control sampling is needed for the Donor, regarding a potential infectious risk). Further, Recipients’ data are anonymized with Hospital attributed bar-codes. None of the authors can access the patient’s file. All needed data are provided anonymously by the physician in charge. Thus, this study is completely anonymized. This procedure protects the anonymity, according to the French Regulation (CNIL).

## Author Contributions

The authors declare no competing financial interests. PD designed and performed the research and analyzed data. FC designed the research, analyzed data, and wrote the paper. B Payrastre, SS, and NB analyzed data and contributed to writing the paper. C-AA and M-AE performed the research and analyzed data. RP, AM, and BPozzetto analyzed data and contributed to writing the paper. OG designed the research, analyzed data, wrote the paper, and supervised the entire project. HH-C designed and performed the research, analyzed data, contributed to writing the paper, and supervised the entire project.

## Conflict of Interest Statement

The authors declare no competing financial interests and no conflicts of interest regarding this study.
