# Supplementary material for: NF-κB Links TLR2 and PAR1 to Soluble Immunomodulator Factor Secretion in Human Platelets
Source: Front Immunol. 2017 Feb 6;8:85. doi: 10.3389/fimmu.2017.00085 (PMC5292648; doi:10.3389/fimmu.2017.00085)
Supplement: Supplementary file 1 [file Image_1.PDF]

Supplemental Figure 1: TLR2 expression in human platelets. (A) The surface expression of TLR2 was detected by antibody labeling and flow cytometry analysis after gating for CD41$^+$. One representative experiment out of 10 is shown. **(B)** Summary of the flow cytometry analysis of TLR2 expression on CD41$^+$ platelets. The mean percentage of CD41$^+$ platelets positive for TLR2 expression is shown (mean ± SD from ten independent experiments). **(C)** Western Blot analysis showing the expression of TLR2, MyD88, NF-κB, Histone H3 and α-tubulin proteins in human platelets. One representative experiment out of five is shown. α-tubulin was used as the loading control.

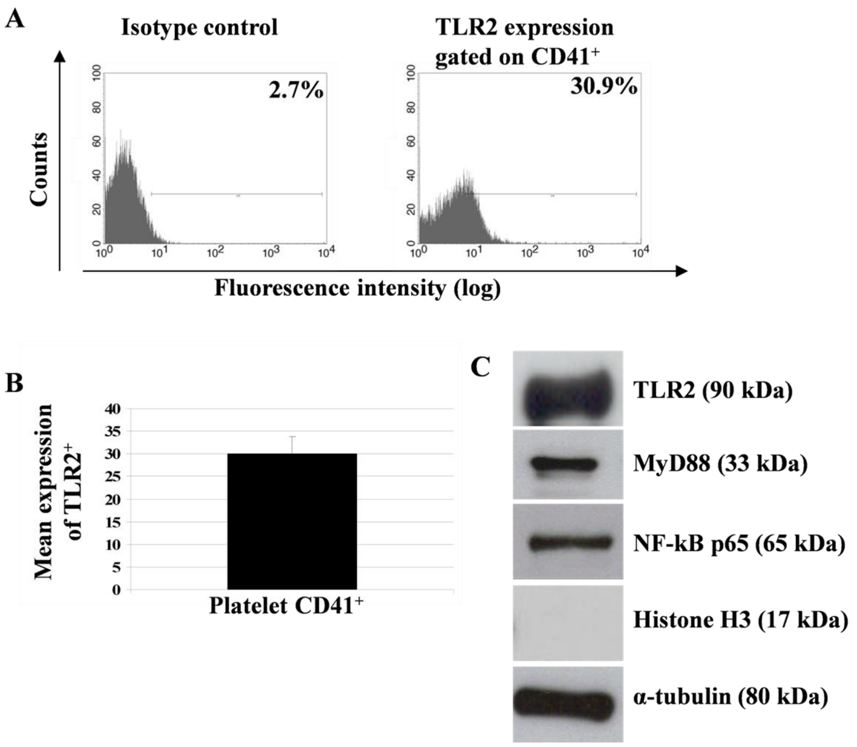

.
