# Supplementary material for: NF-κB Links TLR2 and PAR1 to Soluble Immunomodulator Factor Secretion in Human Platelets
Source: Front Immunol. 2017 Feb 6;8:85. doi: 10.3389/fimmu.2017.00085 (PMC5292648; doi:10.3389/fimmu.2017.00085)

Supplemental Figure 2: Platelet sCD62P (A) and RANTES (B) released by platelets stimulated by Pam3CSK4. A titration and time course was performed in platelets from 5 controls (measured in triplicate) to determine the optimal concentration of Pam3CSK4 for platelet activation, using sCD62P and RANTES release as an end point. The optimal concentration of Pam3CSK4 required to achieve maximal activation (sCD62P and RANTES release) was demonstrated at 2 hours of TLR2 stimulation by its ligand Pam3CSK4 at 100 µg/ml. *P < 0.05 (Mann-Whitney U test; stimuli versus unstimulated).

A)

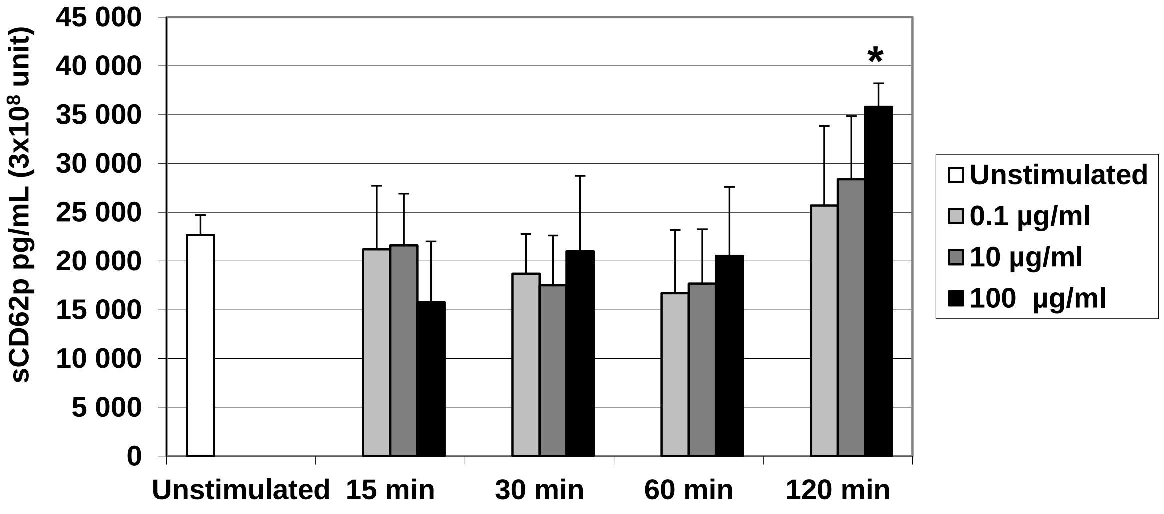

B)

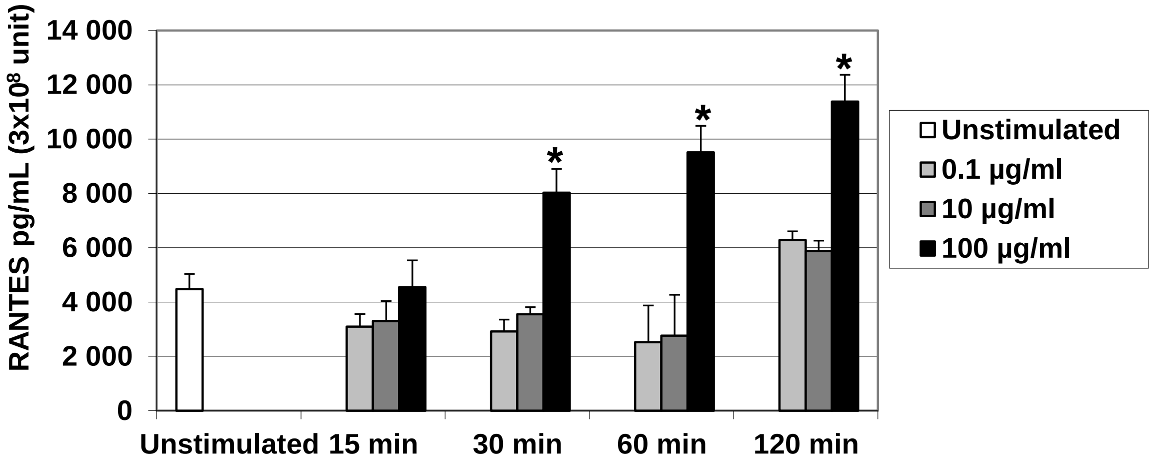

Supplement: Supplementary file 2 [file Image_2.PDF]
