# Supplementary material for: NF-κB Links TLR2 and PAR1 to Soluble Immunomodulator Factor Secretion in Human Platelets
Source: Front Immunol. 2017 Feb 6;8:85. doi: 10.3389/fimmu.2017.00085 (PMC5292648; doi:10.3389/fimmu.2017.00085)

Supplemental Figure 3: CD62P expression on platelets. CD62P was detected by antibody labeling and flow cytometric analysis after gating for CD41$^+$. Summary of flow cytometric analyses of CD62P expression by CD41$^+$ platelets with (w) or without (w/o) anti-CD32. The mean percentage of CD41$^+$ platelets positive for CD62P expression is shown (mean ± SD from five independent experiments).

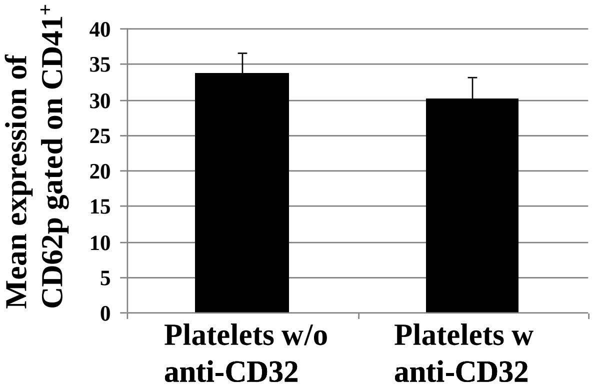

Supplement: Supplementary file 3 [file Image_3.PDF]
