# Supplementary material for: NF-κB Links TLR2 and PAR1 to Soluble Immunomodulator Factor Secretion in Human Platelets
Source: Front Immunol. 2017 Feb 6;8:85. doi: 10.3389/fimmu.2017.00085 (PMC5292648; doi:10.3389/fimmu.2017.00085)

Supplemental Figure 4: Serotonin, sCD62P, RANTES and sCD40L released by unstimulated platelets or platelets incubated with an irrelevant MoAbs IgG2a (isotype-matched irrelevant antibody serves as a control for anti-human TLR2 blocking MoAb). The levels of serotonin, sCD62P, RANTES, and sCD40L were quantified by ELISA. Background levels were subtracted and the data are mean ± SD (n = 4 experiments, measured in triplicate) and are expressed as pg/ml ($3x10^8$ unit - sCD62P, RANTES and sCD40L) or as µg/ml ($3x10^8$ unit – serotonin).

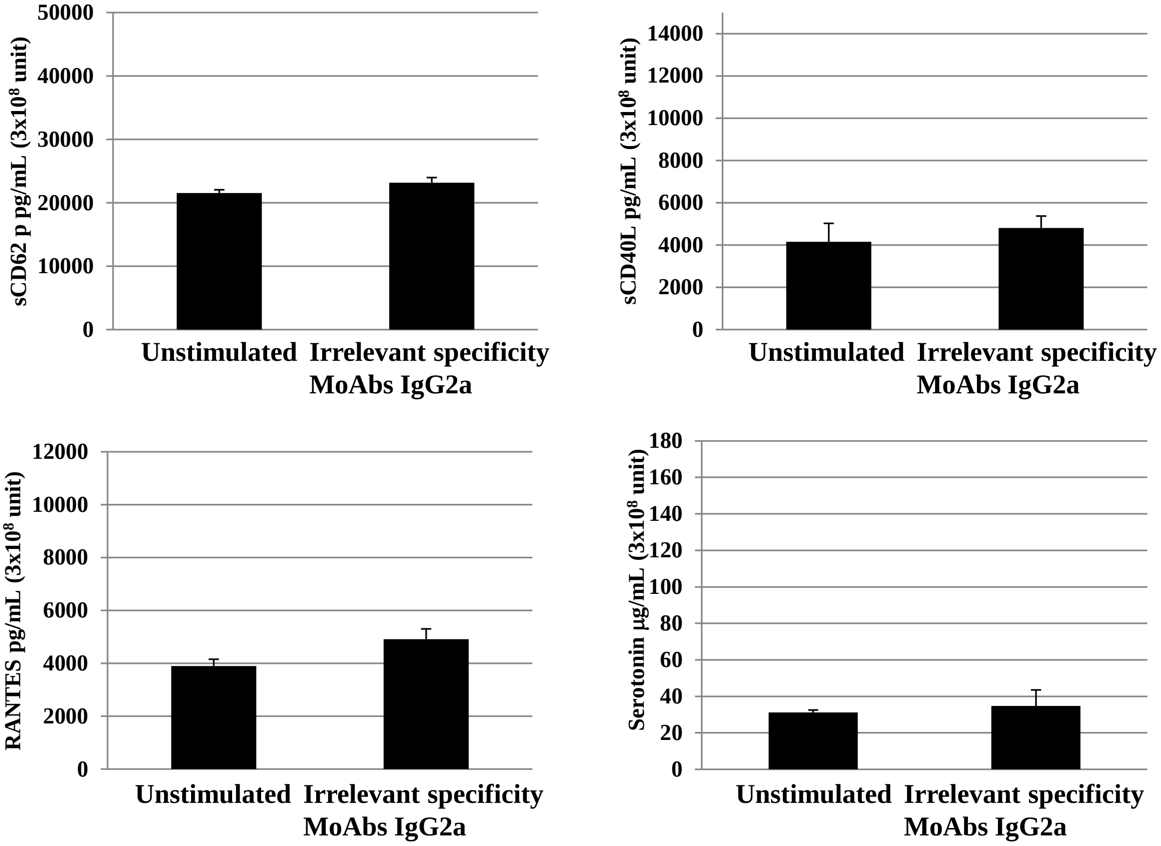

Supplement: Supplementary file 4 [file Image_4.PDF]
