# Supplementary material for: NF-κB Links TLR2 and PAR1 to Soluble Immunomodulator Factor Secretion in Human Platelets
Source: Front Immunol. 2017 Feb 6;8:85. doi: 10.3389/fimmu.2017.00085 (PMC5292648; doi:10.3389/fimmu.2017.00085)
Supplement: Supplementary file 5 [file Image_5.PDF]

Supplemental Figure 5: Platelet sCD62P release following TLR2, 4, 7 and 9-stimulation. Fold increase of Platelet sCD62p stimulated or not with TRAP (50 µg/mL), dsDNA-EC (TLR9 ligand – 10 µg/mL), Gardiquimod (TLR7 ligand – 10 µg/mL), E. coli LPS (TLR4 ligand – 10 µg/mL) or Pam3CSK4 (TLR2 ligand – 100 µg/mL). (n = 5 experiments, measured in triplicate)

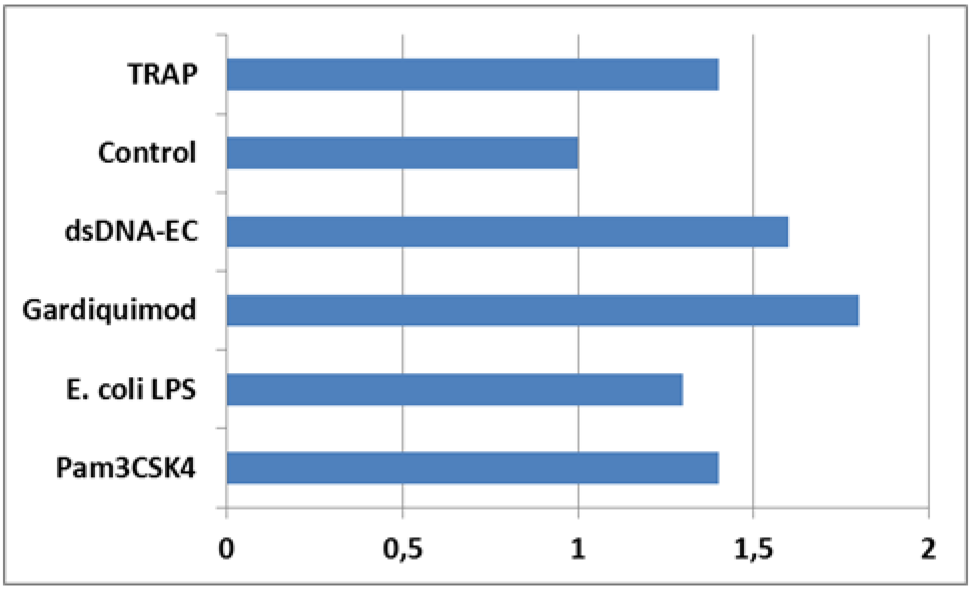

Fold change in sCD62P release
