# Supplementary material for: NF-κB Links TLR2 and PAR1 to Soluble Immunomodulator Factor Secretion in Human Platelets
Source: Front Immunol. 2017 Feb 6;8:85. doi: 10.3389/fimmu.2017.00085 (PMC5292648; doi:10.3389/fimmu.2017.00085)

Supplemental Figure 6: Platelets release soluble PF4. Platelet PF4 is released by platelets stimulated by TRAP (0 to 100 µg/mL - A) or Pam3CSK4 (0 to 100 µg/mL - B) during 120 minutes. The levels of PF4 was quantified by ELISA (measured in triplicate). Background levels were subtracted from the values shown. Data are mean ± SD (n = 10 experiments, measured in triplicate) and are expressed as pg/ml (3x108 unit). *P<0.05 (Mann-Whitney U test; stimuli versus unstimulated).

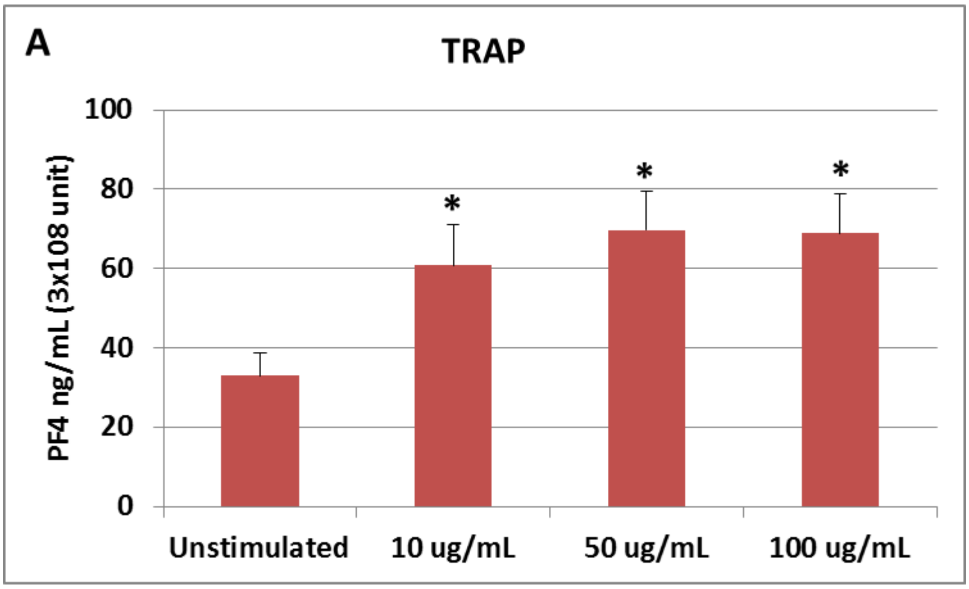

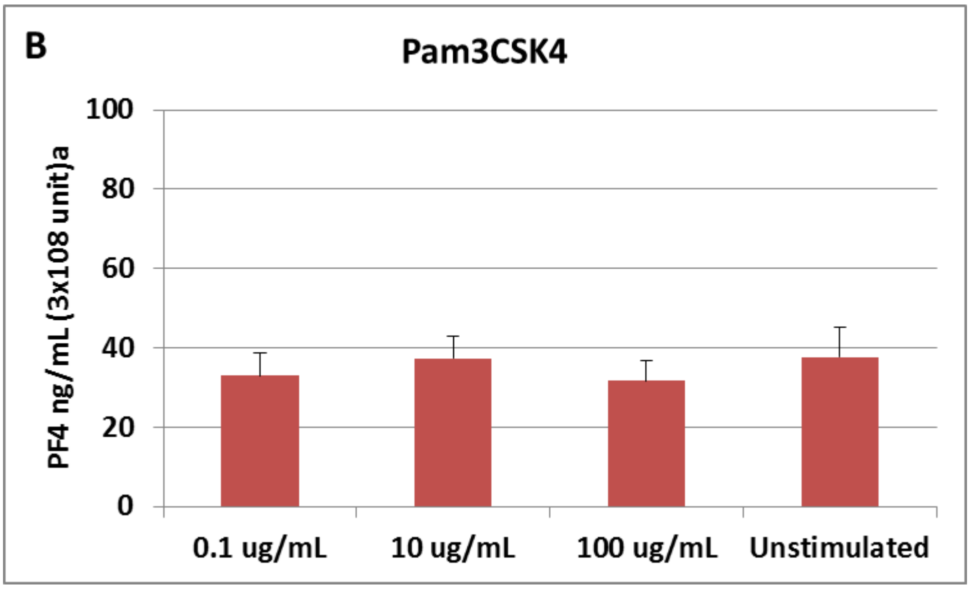

Supplement: Supplementary file 6 [file Image_6.PDF]
